# Associations Between the Big Five Personality Traits and the Non-Medical Use of Prescription Drugs for Cognitive Enhancement

**DOI:** 10.3389/fpsyg.2015.01971

**Published:** 2016-01-05

**Authors:** Sebastian Sattler, Reinhard Schunck

**Affiliations:** ^1^Institute of Sociology and Social Psychology, University of CologneCologne, Germany; ^2^Faculty of Sociology, Bielefeld UniversityBielefeld, Germany

**Keywords:** pharmaceutical cognitive enhancement, non-medical use of prescription drugs, substance abuse, drug misuse, five-factor model, personality traits

## Abstract

While the number of studies of the non-medical use of prescription drugs to augment cognitive functions is growing steadily, psychological factors that can potentially help explain variance in such pharmaceutical cognitive enhancement (CE) behavior are often neglected in research. This study investigates the association between the Big Five personality traits and a retrospective (prior CE-drug use) as well as a prospective (willingness to use CE drugs) measure of taking prescription drugs with the purpose of augmenting one's cognitive functions (e.g., concentration, memory, or vigilance) without medical necessity. We use data from a large representative survey of German employees (*N* = 6454, response rate = 29.8%). The Five Factor Model (FFM) of Personality was measured with a short version of the Big Five Personality Traits Inventory (BFI-S), which includes: openness to experience, conscientiousness, extraversion, agreeableness, and neuroticism. Together with this, demographic variables such as gender, age, education, and income were used as potential confounders in multiple logistic regression models. Our results show a 2.96% lifetime prevalence of CE-drug use and a 10.45% willingness to (re)use such drugs in the future. We found that less conscientious and more neurotic respondents have a higher probability of prior CE-drug use and a greater willingness to use CE drugs in the future. No significant effects were found for openness, extraversion, or agreeableness. Prior CE-drug use was strongly associated with a greater willingness to take such drugs in the future. This study shows that specific personality traits are not only associated with prior enhancement behavior, but also affect the willingness to (re)use such drugs. It helps increase understanding of the risk factors of CE-drug use, which is a health-related behavior that can entail severe side-effects for consumers. The knowledge gathered can thus help improve interventions aimed at minimizing health problems.

## Introduction

Personality traits, which can be described as differences between individuals regarding their behavior, thoughts, and feelings, can be seen as relatively stable in different situations and over time (Caspi, 1998; McCrae and Costa, 2008; Specht et al., 2014). These traits are important predictors of numerous personal, interpersonal, and social/institutional outcomes (Booth-Kewley and Vickers, 1994; Soldz and Vaillant, 1999). Among these are, for instance, happiness, physical and psychological health, longevity, criminal activity, and occupational choices (Booth-Kewley and Vickers, 1994; Ozer and Benet-Martinez, 2006; John et al., 2008). Personality traits (e.g., sensation seeking, neuroticism, impulsivity, anxiety) also seem to be variously associated with the use of different classes of substances and consumption intentions and therefore with risky health behavior that can have deleterious health consequences later in life (e.g., Herman-Stahl et al., 2006; Terracciano et al., 2008; Weyandt et al., 2009; Atherton et al., 2014; N'Goran et al., 2014; Maier et al., 2015).

In this study, we focus on the relationship between personality traits and the non-medical use of prescription drugs (e.g., methylphenidate, modafinil, donepezil) with the subjective aim of augmenting one's cognition (Glannon, 2008; Repantis et al., 2010; Smith and Farah, 2011). These drugs are usually prescribed to treat medical conditions, e.g., attention deficit disorder, narcolepsy, dementia, or Alzheimer's disease. This kind of cognitive enhancement (CE) can be defined as the intended or expected improvement of cognitive functions in healthy individuals in order to augment concentration, vigilance, memory, wakefulness, etc. (e.g., Bostrom and Sandberg, 2009; Repantis et al., 2010; Sattler and Wiegel, 2013)^1^. Given that clinical studies show that the effects of CE with current drugs are limited and sometimes even detrimental (Glannon, 2008; Repantis et al., 2010; Smith and Farah, 2011; Ragan et al., 2013), expectations regarding effectiveness seem often to be exaggerated (Repantis et al., 2010) while at the same time there are also potential risks in terms of side-effects and long-term health consequences (Sussman et al., 2006; Maher, 2008; Winder-Rhodes et al., 2010; Ragan et al., 2013). Beside these risks, the ethical debate about CE-drug use discusses several other potential negative consequences such as whether it undermines authenticity, amounts to cheating/is unfair, increases social inequality, results in direct or indirect coercion to also use such drugs, can burden the health care system, and can result in the involvement of the criminal justice system (Glannon, 2008; Greely et al., 2008; Bostrom and Sandberg, 2009; McLarnon et al., 2012; Dubljević et al., 2014; Sattler, in press).

Despite the possible detrimental effects and (long term) side effects of CE (Sussman et al., 2006; Glannon, 2008; Maher, 2008; Repantis et al., 2010; Winder-Rhodes et al., 2010; Smith and Farah, 2011; Ragan et al., 2013), healthy individuals take such medication for enhancement purposes. In addition to several studies questioning students about their CE use (Middendorff et al., 2012; Maier et al., 2013; Sattler and Wiegel, 2013; Wolff and Brand, 2013; Singh et al., 2014), only a few have surveyed the general population including the working population or parts of it. An informal survey among 1400 readers of the magazine *Nature* reported that 20% have already used such drugs for non-medical reasons to improve concentration, focus, or memory; these include methylphenidate (like Ritalin), modafinil (like Provigil), beta blockers (like propranolol), and/or others (Maher, 2008). The reported 12-month prevalence in a representative German study was 1.5% for drugs used to increase cognition and/or mood including prescription drugs (e.g., drugs counteracting depression such as fluoxetin and/or beta blockers) and illicit drugs (i.e., chemically synthesized stimulants such as amphetamines; Hoebel et al., 2011). A German health insurance company's survey of employees covered under its plans found that 4.7% used such drugs (i.e., stimulants such as methylphenidate, antidementives such as donepezil, and/or antidepressants such as fluoxetin) during their lifetime to enhance cognition and/or mood (DAK Gesundheitsreport, 2009) in a follow-up study prevalence increased to 6.7%, whereby 3.3% used drugs for CE (Marschall et al., 2015). Among German university teachers fewer than 1% reported prior non-medical use of prescription drugs to enhance cognitive performance (without specifying drugs or drug classes); however, more than 10% were willing to use such drugs in the future (Wiegel et al., 2015). This elevated willingness could turn into behavior under certain conditions, e.g., improved benefit-risk ratio or easier access (Singh et al., 2014; Wiegel et al., 2015), which would contribute to the predicted trend of increased CE-drug use.

Existing prevalence estimates are very heterogeneous, however. This is mainly due to inconsistent methods and measures across studies (Smith and Farah, 2011; Ragan et al., 2013; Ford and Ong, 2014; Maier et al., 2015; Sattler, in press). Most studies are not based on probability samples, but instead use small-scale samples, special populations, or combine prescription and illicit drugs or mood and CE into a single category. Thus, to get better estimates of the prevalence of CE-drug use, large-scale population-based probability samples have been strongly recommended (Hoebel et al., 2011; Mache et al., 2012; Sattler et al., 2013a; Fitz et al., 2014; Sattler, in press). Given the potential negative consequences of CE-drug use mentioned above, more empirical data about prevalence are needed on a regular basis for decision-making about the regulation of these drugs (Ragan et al., 2013; Maier and Schaub, 2015) to be better informed and also because of the assumption that the spread of CE drugs on the world market and on the Internet can scarcely be stopped (Sahakian and Morein-Zamir, 2007). More importantly, however, it is necessary to develop a better understanding of the antecedents to CE as a risky health-related behavior in order to inform prevention policies and to develop interventions for reducing its potential negative consequences (Booth-Kewley and Vickers, 1994; Terracciano et al., 2008).

While we can observe an increase in the number of studies that explore social, personal, and the characteristics of the substances as correlates of CE-drug use and willingness to use CE (Sattler and Wiegel, 2013; Wolff and Brand, 2013; Sattler et al., 2013a,b; Dubljević et al., 2014; Ford and Ong, 2014; Singh et al., 2014; Wolff et al., 2014; Maier et al., 2015; Wiegel et al., 2015), we still know very little about the role of psychological variables in relation to CE, in particular how personality characteristics affect CE-drug use. Accordingly, researchers have called for more studies on the effects of psychological variables (Quednow, 2010; Schelle et al., 2014; Wolff et al., 2014; Ponnet et al., 2015). Previous studies, for example, found that high achievement motivation (Franke et al., 2012), the inclination to procrastinate (Sattler et al., 2014; Ponnet et al., 2015), risk attitudes (Sattler and Wiegel, 2013; Sattler et al., 2014), stress (Wolff and Brand, 2013; Wiegel et al., 2015), high pressure to perform (Franke et al., 2013), cognitive test anxiety (Sattler and Wiegel, 2013; Sattler et al., 2014), trait impulsivity (Maier et al., 2015), Machiavellianism (Maier et al., 2015), novelty seeking (Maier et al., 2015), lower cognitive empathy (Maier et al., 2015), and burnout (Wolff et al., 2014) were positively associated with CE-drug use or willingness to use. Our study aims at increasing our knowledge about the antecedents of CE-drug use by further investigating the relationship between personality characteristics and CE. Since it is advisable to employ multidimensional systems of personality with a well-validated factor structure (Sher et al., 2000), we use the Five-Factor Model (FFM) of personality, which is a widely used and dominant paradigm in personality psychology (Costa and McCrae, 1995; Ozer and Benet-Martinez, 2006; Terracciano et al., 2008). It covers the five major traits: openness to experience, conscientiousness, extraversion, agreeableness, and neuroticism (Costa and McCrae, 1995; Terracciano et al., 2008).

To our knowledge, this is the first study using a representative large-scale sample of German employees to investigate how personality traits are associated with CE. The only existing study that has examined the Big Five personality traits and a broad retrospective measure of CE (by combining certain prescription, non-prescription, and illegal drugs) with bivariate analyses is based on a student sample; the results are described below (Middendorff et al., 2012). Given this lack of research, our study can be informed by studies on the Big Five personality traits and other forms of substance use and misuse such as tobacco, alcohol, prescription stimulants, marihuana, etc.

*Openness to experience* can be described as a person's appreciation of new experiences and stimulation due to being imaginative, creative, unconventional, and emotionally as well as aesthetically sensitive (Caspi et al., 2005; John et al., 2008; Terracciano et al., 2008). It has been assumed that this willingness to engage in new experiences is a risk factor for the non-medical use of prescription drugs (Benotsch et al., 2013) and/or for detrimental substance use (Turiano et al., 2012). Specifically for CE, a greater openness to experiences has been presumed to promote CE-drug use due to higher eagerness to experiment (Middendorff et al., 2012). Several studies corroborate this assumption by showing that, for example, higher openness was positively associated with marijuana use (Terracciano et al., 2008) or illegal drug use in general (Turiano et al., 2012), opioid dependency (Kornør and Nordvik, 2007), cigarette smoking (Turiano et al., 2012), problem drinking (Turiano et al., 2012), a broader measure of substance-related risk-taking (defined as drinking, driving after drinking, smoking tobacco; Booth-Kewley and Vickers, 1994), substance-use disorder (Trull and Sher, 1994), and the non-medical use of prescription drugs (Benotsch et al., 2013). However, the only existing study of a set of different CE drugs found no effect (Middendorff et al., 2012).

*Conscientiousness* refers to an ability to control behavioral and cognitive impulses “that facilitates task- and goal-directed behavior, such as thinking before acting, delaying gratification, following norms and rules, and planning, organizing, and prioritizing tasks” (John et al., 2008, p. 138; cf. Caspi et al., 2005; Terracciano et al., 2008). It is seen as a protective factor against the non-medical use of prescription drugs (Benotsch et al., 2013) and the latter's specific form of CE-drug use (Middendorff et al., 2012), but also more generally against the detrimental use of other substances (Turiano et al., 2012). High levels of conscientiousness are assumed to play an important self-regulatory role and are associated with discipline and persistence and thus with disregarding the immediate gratification of health-damaging behaviors in order to obtain future, long-term outcomes (such as long-term health) “instead of” positive future, long-term health (Middendorff et al., 2012; Turiano et al., 2012; Benotsch et al., 2013). One argument is that since a reduced ability to engage in systematic and organized behavior has detrimental effects on learning, CE-drug use might be a means to compensate for these effects (Middendorff et al., 2012). Prior research has shown that procrastination, which can be seen as an example of this reduced ability, predicts a willingness to use CE drugs (Sattler et al., 2014). The only study on CE-drug use and conscientiousness thus far has also described a negative association between the two, namely that more conscientious students less were less likely to report using several drugs for purposes of CE (Middendorff et al., 2012). Furthermore, for a majority of respondents, CE-drug use violates social norms (e.g., fairness; Sattler et al., 2013b; Dubljević et al., 2014; Schelle et al., 2014; Wiegel et al., 2015; Sattler, in press) and since conscientious individuals tend to follow norms and rules, they might be less likely to use such drugs. Research on other types of substance use also found that increased conscientiousness is associated with a lower incidence of cigarette smoking (Terracciano et al., 2008; Turiano et al., 2012), of non-medical use of prescription drugs (Turiano et al., 2012; Benotsch et al., 2013), of use of illegal drugs such as marijuana, cocaine, and hallucinogens/lysergic acid diethylamide (LSD) (Terracciano et al., 2008; Turiano et al., 2012), of alcohol consumption (Malouff et al., 2007; Turiano et al., 2012), of substance use disorders (Trull and Sher, 1994), and specifically of opioid dependency (Kornør and Nordvik, 2007) as well as lower more general measures of the use of substances including cigarettes, alcohol, and recreational drugs (Atherton et al., 2014; cf., Lackner et al., 2013).

*Extraversion* reflects an energetic approach toward the world and can be understood as a person's tendency to be outgoing, expressive, active, energetic, assertive, cheerful, sociable, and in search of stimulation (Caspi et al., 2005; John et al., 2008; Terracciano et al., 2008). Some researchers have assumed neither an association between extraversion and CE-drug use specifically (Middendorff et al., 2012), nor with substance use in general (Turiano et al., 2012). However, several studies disprove this assumption. For example, it has been found that higher extraversion correlates with increased alcohol consumption (Turiano et al., 2012), more tolerant attitudes toward substance use (Francis, 1996), and more frequent use of substances including cigarettes, alcohol, and recreational drugs (Atherton et al., 2014). Yet, the association between extraversion and substance use may depend on the specific substance in question. Studies found that extraversion was higher in smokers as well as marijuana and cocaine/heroin users (Terracciano et al., 2008) but lower for opioid dependents (Kornør and Nordvik, 2007). A broad CE-measure, however, was uncorrelated with extraversion.

*Agreeableness* can be defined as a person's pro-social and communal orientation and includes a person's tendency to be altruistic, trustworthy, cooperative, considerate, empathic, polite, and modest (Caspi et al., 2005; John et al., 2008; Terracciano et al., 2008). While it has been assumed that agreeableness associates negatively with substance use (Booth-Kewley and Vickers, 1994; Turiano et al., 2012), for CE-drug use specifically it has been predicted that there would be no effect (Middendorff et al., 2012). This latter assumption has been supported by one study (Middendorff et al., 2012). For other substances, agreeableness seems to have a protecting effect, since more agreeable persons report lower marijuana use (Terracciano et al., 2008), alcohol consumption (Malouff et al., 2007; Turiano et al., 2012), non-medical use of prescription drugs (Benotsch et al., 2013), polydrug abuse (Lackner et al., 2013), and alcohol dependency (Kornør and Nordvik, 2007).

*Neuroticism* includes feelings such as anxiety, nervousness, sadness, and depression and thus reflects a tendency to experience negative emotions (Caspi et al., 2005; John et al., 2008; Terracciano et al., 2008). Neuroticism is seen as a risk factor for CE-drug use (Middendorff et al., 2012) and more generally for the non-medical use of prescription drugs as well (Benotsch et al., 2013), but also for the use of other substances (Turiano et al., 2012). According to the self-medication hypothesis (Khantzian, 1997; West, 2005), individuals use drugs and may become dependent on them because they are vulnerable to stress, emotionally unstable, and thus may use CE to cope with emotional distress (Kornør and Nordvik, 2007; Benotsch et al., 2013). Contrary to these assumptions, neurotic individuals might also be more anxious about the potential side effects of CE-drug use, which might inhibit their use. However, several studies have corroborated that more neurotic individuals report higher use of a variety of substances, e.g., a broad CE-measure (Middendorff et al., 2012), alcohol consumption (Malouff et al., 2007; Turiano et al., 2012), cigarette smoking (Terracciano et al., 2008; Turiano et al., 2012), illegal drug use (Turiano et al., 2012) including cocaine and heroin (Benotsch et al., 2013), and prescription drug use especially anxiolytics and sedatives (Turiano et al., 2012; Benotsch et al., 2013); they are also more likely to report polydrug addiction (Lackner et al., 2013) and drug use disorder (Sher et al., 2000).

Due to scarce data on CE-drug use and the FFM and given the often inconsistent correlations between Big Five dimensions and many kinds of substance use and misuse from heterogeneous studies, there is a clear need for more research in this field (e.g., Booth-Kewley and Vickers, 1994; Francis, 1996; Malouff et al., 2007; Terracciano et al., 2008; Turiano et al., 2012; Benotsch et al., 2013; Lackner et al., 2013; Atherton et al., 2014).

By using data from a representative, large-scale random sample of German employees, this study aims at expanding our understanding of the prevalence of prior non-medical use of prescription drugs (a retrospective measure) and the willingness to use such drugs in the future (a prospective measure) with the subjective purpose of augmenting one's cognitive performance (by improving functions such as concentration, memory, or vigilance) as well as the association between the prevalence and willingness and the FFM traits. On a more general level, the study also adds to our understanding of the effect of personality traits on substance use: although mainly illicit drugs as well as substances such as alcohol and nicotine have been investigated in this regard, the non-medical use of prescription drugs has less frequently been the subject of research (Benotsch et al., 2013; N'Goran et al., 2014). Moreover, several studies on substance use and personality traits have investigated only one or a subset of traits of the FFM, while the present study investigates all five domains (Booth-Kewley and Vickers, 1994; Turiano et al., 2012; N'Goran et al., 2014).

## Methods

### Research design and data

#### Data

The data for this study are based on the first wave of the B3 Linked Employer-Employee Panel Survey (LEEP-B3) (Diewald et al., 2014). For the purpose at hand, we use the employee-survey of the LEEP-B3 data, which were collected as computer-assisted telephone interviews (CATI) in 2012–13 in Germany. Prior to the interviews selected participants were informed by a cover letter about the subject of the study, the voluntariness of their participation, their anonymity, and the confidentiality of all their answers. These issues were explained again during the first telephone contact. The underlying population comprises all employees in Germany who are subject to social security contributions, which applies to the majority of German employees—excluding only self-employed, marginally employed, apprentices, and civil servants. The net sample comprised 21,678 eligible respondents. The response rate was 29.77%, which leads to a total sample of 6454 (Diewald et al., 2014). Multivariate selectivity analyses comparing the sample to the underlying population using German registry data indicate that the LEEP-B3 data represent the underlying population rather well (Diewald et al., 2014). There is some limited selectivity, namely people who are German nationals and work in the “information and communication” sector participated in greater numbers, whereas people with lower levels of education and those working in very large organizations were less likely to participate.

### Ethics statement

In Germany, ethics approval for social science research is not required if research objectives do not investigate issues regulated by law (e.g., the German Medicine Act [Arzneimittelgesetz, AMG], the Medical Devices Act [Medizinproduktegesetz, MGP], the Stem Cell Research Act [Stammzellenforschungsgesetz, StFG], or the Medical Association's Professional Code of Conduct [Berufsordnung der Ärzte]). Since our study had no such objectives, approval was not required. Furthermore, paragraph 28 of the Data Protection Act of North Rhine Westphalia (Datenschutzgesetz Nordrhein-Westfalen, DSG NRW) explains that personal data have to be processed anonymously and that participants' consent is required only when the data are not used anonymously. Since data were collected in cooperation with the federal Institute for Employment Research (Institut für Arbeitsmarkt und Berufsforschung, IAB; Diewald et al., 2014), the study and all procedures were approved by the data security officer of the federal IAB and the Federal Ministry of Labor and Social Affairs (Bundesministerium für Arbeit und Soziales, BAMS). Prior to the interviews the selected participants were informed by a cover letter about the subject of the study. This letter explicitly informed the potential participants of the voluntariness of their participation, their anonymity, and the confidentiality of all their answers. During the first telephone contact, potential participants were again explicitly informed that their participation was voluntary, that all answers would be treated confidentially, and that the data would be anonymized. Thus, the act of participating in the study after receiving all relevant confidentiality information was taken to imply understanding and agreement.

### Measures

#### Prior CE-drug use

We measured prior CE-drug use by asking: “Some people support their cognitive abilities with the help of prescription drugs, though there is no medical need (e.g., for increasing concentration, memory, or vigilance). Have you ever done that?” We provided the following response categories: “no, never” (0); “yes, within the last 30 days” (1); “yes, between the last 30 days and 6 months” (2); “yes, between the last 6 months and 1 year” (3); “yes, more than 1 year ago” (4) (cf. Sattler and Wiegel, 2013; Wiegel et al., 2015). Due to the low prevalence (see Table 1), a dichotomous variable was computed for our multivariate analysis, indicating no use (0) and prior use (1) (cf. Wiegel et al., 2015).

**Table 1:** **Descriptive statistics with non-imputed data**.

	**Mean**	**Standard deviation**	**Min**	**Max**	**Observations**	**Observations with missing values (in %)**
**COGNITIVE ENHANCEMENT**						
Prior CE-drug use^a^	0.03	–	0.00	1.00	6444	10 (0.15)
Willingness to use CE drugs	0.10	–	0.00	1.00	6332	122 (1.89)
**BIG FIVE PERSONALITY TRAITS INVENTORY**						
Openness to experiences	0.00	1.00	−3.76	2.92	6407	47 (0.73)
Conscientiousness	0.00	1.00	−5.89	2.82	6407	47 (0.73)
Extraversion	0.00	1.00	−3.46	2.49	6407	47 (0.73)
Agreeableness	0.00	1.00	−4.84	2.60	6407	47 (0.73)
Neuroticism	0.00	1.00	−3.02	3.46	6407	47 (0.73)
**SOCIO-DEMOGRAPHIC CONTROLS**						
Male	0.53	–	0.00	1.00	6454	0 (0.00)
Age in years	40.63	8.64	19.00	52.00	6454	0 (0.00)
Education in years	14.04	2.83	7.00	18.00	6408	46 (0.71)
Gross monthly earnings in Euro	3766.70	3650.65	13.27	125,000.00	6136	318 (4.93)

*Source: LEEP-B3, own computations*.

a *This category includes 50 (0.78%) respondents indicating CE-drug use within the last 30 days, 24 (0.37%) respondents indicating such usage between the last 30 days and 6 months, 27 (0.42%) respondents indicating such usage between the last 6 months and 1 year and 90 respondents (1.40%) indicating that such usage was more than 1 year ago*.

#### Willingness to use CE drugs

Given that CE-drug use can be described as a relatively new and potentially increasing phenomenon (e.g., Farah et al., 2004; Castaldi et al., 2012), we also assessed the respondents' willingness to use CE drugs, since this can be seen as one method for determining whether the postulated trend exists (Wiegel et al., 2015). Willingness measures are often used in research on the use of (licit and illicit) substances such as tobacco, alcohol, amphetamines, and marijuana, since they are used as proximal antecedents of future behavior (Gibbons et al., 1998a,b; Gerrard et al., 2006). However, an imperfect correlation between this measure and behavior may exist, since behavioral restrictions can change over time (cf. Grasmick and Bursik, 1990). But such measures are assumed to be less sensitive than behavioral measures and thus should result in fewer item-non responses or biased responses (e.g., Gibbons et al., 1998b). The CE-willingness measure was similar to the prior CE-drug use measure. Respondents were asked whether they could imagine using (or reusing) such prescription drugs for CE in the future (cf. Ponnet et al., 2015; Wiegel et al., 2015). Dichotomous response categories were “No, I would not do that under any circumstances” (0) and “Yes, I would do that under certain circumstances” (1) (cf. ZUMA, 1990).

#### Personality traits

We used a short version of the Big Five Personality Traits Inventory (BFI-S) (Gerlitz and Schupp, 2005; Dehne and Schupp, 2007; Hahn et al., 2012) to assess the components of the FFM of personality (e.g., Costa and McCrae, 1995). Each of the five factors (openness to experience, conscientiousness, extraversion, agreeableness, and neuroticism) were measured by three items on five-point scales ranging from “I agree entirely” (1) to “I do not agree at all” (5). The items have been reverse-coded so that higher values indicate stronger agreement with the underlying factor (see Table S1). With regard to reliability, the BFI-S is a reasonable instrument for measuring the FFM in large, multi-purpose surveys (Lang et al., 2011). Similar to prior research (Dehne and Schupp, 2007), reliability analysis of the scales showed moderate internal consistencies: openness to experiences (artistic experiences, ideas, active imagination, α = 0.53), extraversion (talkative, sociable, reserved, α = 0.66), conscientiousness (efficient, thorough job, lazy, α = 0.55), agreeableness (forgiving, kind, rude, α = 0.45), and neuroticism (worried, nervous, relaxed, α = 0.54). However, since each item is supposed to measure a distinct facet within each dimension, the relatively low alpha values can be seen as an indication of the distinctness of the underlying facets (Rammstedt, 2010). We used explanatory factor analysis with varimax rotation to extract the five factors from the BFI-S (see Table S1). All items loaded substantially on the respective factor (openness with a mean factor loading of 0.68; extraversion with a mean factor loading of 0.76; conscientiousness with a mean factor loading of 0.69; agreeableness with a mean factor loading of 0.66; and neuroticism with a mean factor loading of 0.71) and showed low secondary loadings on other factors (mean secondary loading = 0.07).

#### Demographic variables

Women were coded “0” and men “1” (see Table 1 for this and other descriptive statistics). We also assessed gender (female = 0 and male = 1), age, education in years (each educational degree is assigned the average duration it takes to obtain), and personal gross monthly earnings in Euro.

#### Missing values

The proportion of missing values is generally low (see Table 1). The highest proportion of missings can be found with earnings (4.93%, *n* = 318). Missing values were imputed using multivariate imputation by chained equations (MICE) (Azur et al., 2011; White et al., 2011) with 20 data sets. The following multivariate analyses are based on the imputed data sets, but all analyses have also been carried out using the unimputed data set (see Tables S2, S3).

### Statistical analysis

We used logistic regression models to test how the dependent variables covary with the independent variables. We report odds ratios (OR). ORs greater than 1 indicate positives effects of the independent variables on the respective dependent variable, while ORs lower than 1 indicate a negative effect, and ORs equal to 1 indicate no effect. The reported *p*-values are based on robust standard errors.

## Results

### Prior CE-drug use

Our descriptive results (based on non-imputed data) show that approximately 97.04% (*n* = 6253) of the respondents report that they have never used prescription medication non-medically to support their cognitive abilities (see Table 1), while 2.96% (*n* = 191) reported such CE-drug use during their lifetime (see Table 1). In particular, 0.78% (*n* = 50) reported having used such drugs within the last 30 days, 0.37% (*n* = 24) between the last 30 days and 6 months, 0.42% (*n* = 27) between the last 6 months and 1 year, and 1.40% (*n* = 90) reported having used such drugs over a year ago. Our multivariate analysis (based on imputed data) focuses on the lifetime prevalence of CE-drug use only (see Methods section). Results show an OR of 0.774 (*p* < 0.001) in Model 1 in Table 2, which indicates a significant negative association between conscientiousness and prior CE-drug use. Thus, more conscientious respondents had a lower probability of prior CE-drug use. Moreover, we found a positive association between neuroticism and CE-drug use (*p* < 0.001). No significant associations were found for openness to experiences (*p* = 0.101), extraversion (*p* = 0.416), or agreeableness (*p* = 0.376). Prior use of CE drugs did not significantly vary with the socio-demographic controls gender (*p* = 0.865), age (*p* = 0.811), and earnings (*p* = 0.404)^2^. Education, however, was found to have a negative association with prior CE-drug use (*p* = 0.049).

**Table 2:** **Logistic regression models to assess associations of the BFI-S and socio-demographic controls with prior CE-drug use (Model 1) and the willingness to use CE drugs (Model 2 and 3) with imputed data (Number of imputations = 20, Number of observations = 6454)**.

	**Model 1 Prior CE-drug use**		**Model 2 Willingness to use CE drugs**		**Model 3 Willingness to use CE drugs**	
	**OR**	**95% CI**	**OR**	**95% CI**	**OR**	**95% CI**
Openness to experience	1.129	[0.977, 1.305]	1.018	[0.935, 1.107]	0.999	[0.914, 1.092]
Conscientiousness	0.774^***^	[0.674, 0.888]	0.810^***^	[0.748, 0.876]	0.831^***^	[0.766, 0.903]
Extraversion	1.061	[0.919, 1.225]	1.067	[0.981, 1.159]	1.062	[0.975, 1.158]
Agreeableness	0.941	[0.822, 1.077]	0.931	[0.859, 1.009]	0.934	[0.858, 1.017]
Neuroticism	1.352^***^	[1.154, 1.584]	1.303^***^	[1.197, 1.418]	1.264^***^	[1.158, 1.379]
Male	0.865	[0.628, 1.192]	0.798^*^	[0.669, 0.951]	0.802^*^	[0.667, 0.964]
Age in years	0.998	[0.981, 1.015]	1.005	[0.995, 1.015]	1.006	[0.996, 1.016]
Education in years	0.944^*^	[0.891, 1.000]	0.988	[0.958, 1.018]	0.997	[0.966, 1.029]
Gross monthly earnings in Euro	1.000	[1.000, 1.000]	1.000	[1.000, 1.000]	1.000	[1.000, 1.000]
Prior CE-drug use					17.320^***^	[12.608, 23.792]
Constant	0.080^***^	[0.025, 0.256]	0.120^***^	[0.066, 0.220]	0.083^***^	[0.045, 0.156]
Log pseudolikelihood	−840.449		−2114.087		−1955.289	
Pseudo *R*²	0.026		0.021		0.094	

*Source: LEEP-B3, own computations*.

*OR = Odds Ratios. CI = 95% confidence intervals in parentheses (based on robust standard errors). Log pseudolikelihood and Pseudo R^2^ are averaged across imputed datasets*.

* *p < 0.05*,

^**^p < 0.01,

*** *p < 0.001*.

### Willingness to use CE drugs

10.45% (*n* = 662, based on non-imputed data) of the respondents reported being willing to consume CE drugs in the future, while the remaining 89.55% (*n* = 5.670) indicated that they would never use such drugs (see Table 1). Multivariate analysis (based on imputed data) shows that the willingness to use CE drugs decreased if respondents showed stronger tendencies toward conscientiousness (*p* < 0.001) and it increased if respondents reported higher levels of neuroticism (*p* < 0.001) (see Model 2 in Table 2). Again, the effects for the domains openness to experience (*p* = 0.687), extraversion (*p* = 0.128), and agreeableness (*p* = 0.080) reached no conventional levels of significance. Males were less willing to use CE drugs in the future compared to females (*p* = 0.012). Age (*p* = 0.320), education (*p* = 0.414), and earnings (*p* = 0.848) had no significant effects on the willingness to use CE drugs^3^. Respondents reporting the use of CE drugs in the past were much more willing to consume such drugs in the future compared to those who had never used such drugs (*p* < 0.001; Table 2, Model 3). Finally, we tested whether the effects of the five personality domains and the socio-demographic variables were conditional on prior use by adding interaction terms of these variables with prior use (see Table S3). Results show that no differential effects exist, i.e., the effects of the five personality domains and the socio-demographics on the willingness to use CE drugs do not differ between users and non-users.

**Figure 1:**
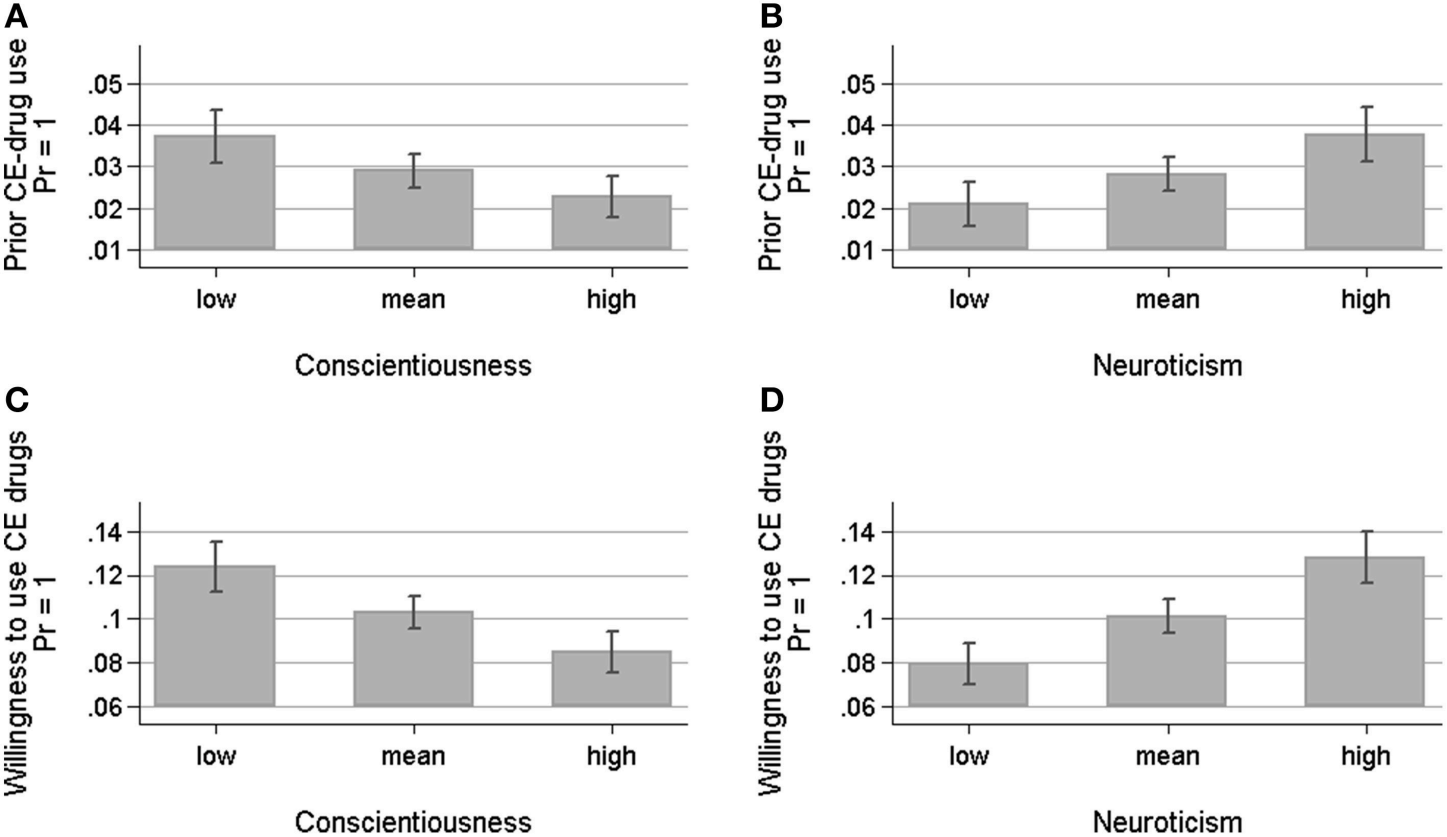
**Predicted probabilities of prior use and willingness (both y-axis) estimated using average marginal effects based on multivariate logistic regression models—error bars represent the 95% confidence interval. (A)** shows that the predicted probability of prior CE-drug use is higher in case of lower conscientiousness [defined by the mean value (M) − 1 standard deviation (SD)] compared to the M and to higher conscientiousness (M + 1 SD), while the probability **(B)** is lower for lower levels of neuroticism (M – 1 SD) compared to the M and to higher neuroticism (M + 1 SD) (based on Model 1, Table 2). **(C,D)** show similar effects for the willingness to use CE drugs (based on Model 2, Table 2).

To facilitate the interpretation, the main results concerning the association between prior CE-drug use and the two personality traits of conscientiousness and neuroticism as well as the association between willingness to use CE drugs and these two traits are displayed in Figure 1. It shows predicted probabilities for both CE-measures using average marginal effects based on the multivariate models in Table 2. The predicted probabilities show that the effects associated with conscientiousness and neuroticism are rather large. The predicted difference between respondents with a low [defined by the mean (M) minus one standard deviation (SD)] and a high level of conscientiousness (M + 1 SD) is 39 percentage points (3.71 vs. 2.26%) in prior use and 31 percentage points regarding willingness to use (12.38 vs. 8.51%) (Figures 1A,C). As regards neuroticism, the predicted difference between respondents with a high level (M + 1 SD) and respondents with a low level (M – 1 SD) of this personality trait is 44 percentage points in prior use (3.76 vs. 2.10%) and 38 percentage points in willingness to use CE drugs (12.78 vs. 7.98%) (Figures 1B,D).

## Discussion

### Summary and interpretation of the results

#### Prevalence of CE-drug use and the willingness to take CE drugs

While there is a fierce debate about whether CE-drug use is already a widespread phenomenon or whether it will be widespread in the future (Sahakian and Morein-Zamir, 2007; Greely et al., 2008; Ragan et al., 2013; Sattler et al., 2013a), limited research has been based on prevalence estimates derived from large-scale random samples beyond student populations (Hoebel et al., 2011; Mache et al., 2012; Sattler et al., 2013a; Fitz et al., 2014; Sattler, in press). We add to this research with data from more than **six** thousand employees in Germany randomly selected for this study (Diewald et al., 2014). We found a lifetime prevalence of nearly 3% for use of prescription drugs for supporting cognitive performance. This figure falls in the range of prevalence estimates of comparable prior studies (DAK Gesundheitsreport, 2009; Hoebel et al., 2011; Marschall et al., 2015) and shows that CE-drug use is already a fact, even if not a general practice, which opposes the current media hype about CE. Still, it has been estimated that more than half a million individuals in Germany have experience with CE-drug use (Kowalski, 2013); it can be presumed that a large number of these continue using (Sattler and Wiegel, 2013; Sattler et al., 2014; Wiegel et al., 2015), risking potential detrimental health effects often with no real effects or even detrimental effects. But the willingness to use such drugs in the future was more than three times greater than lifetime prevalence. More than every 10th respondent indicated such a willingness. A similar difference between prior and potential future use has been found in a study of university teachers (Wiegel et al., 2015). Of course expressed willingness does not necessarily translates into actual behavior, for example due to changes in behavioral restrictions (cf. Grasmick and Bursik, 1990). However, discrepancies between willingness and use of CE drugs may also be explained by other factors. For example, potential users may not yet have experienced a pressing need to take such drugs, but would do so if the need were to occur, or they might not have had the opportunity (e.g., due to lack of access) to convert their interest into use (Singh et al., 2014; Wiegel et al., 2015). But drugs may become more available via the Internet and on the black market (Sahakian and Morein-Zamir, 2007). Potential users may also want to wait until more effective and safer medication is available (DAK Gesundheitsreport, 2009; Franke et al., 2012; Wiegel et al., 2015).

#### Associations between big five personality traits and prior CE-drug use and the willingness to engage in CE-drug use

Only relatively few studies (e.g., Franke et al., 2012, 2013; Sattler and Wiegel, 2013; Wolff and Brand, 2013; Sattler et al., 2014; Wiegel et al., 2015; Maier et al., 2015) have responded to one of the first appeals for investigating how personality relates to CE-drug use (Quednow, 2010); in these the Big Five traits—which represent an important set of traits—have generally been ignored, with the exception of one study among students (Middendorff et al., 2012). By investigating the association between Big Five traits and prior CE-drug use as well as the willingness to use CE drugs in the future, we hope to add to our understanding of how a set of five major personality traits relates to such behavior. This also contributes to the often inconsistent findings between various kinds of substance use and misuse (including tobacco, alcohol, prescription stimulants, marihuana, etc.) and the FFM (e.g., Booth-Kewley and Vickers, 1994; Francis, 1996; Malouff et al., 2007; Terracciano et al., 2008; Turiano et al., 2012; Benotsch et al., 2013; Lackner et al., 2013; Atherton et al., 2014).

Similarly to a study of students using a broad retrospective measure of CE that combines certain prescription, non-prescription, and illegal drugs (Middendorff et al., 2012), we found that openness to experience was unrelated to both prior CE-drug use and the willingness measure. This, however, contradicts the assumption that the tendency to engage in new experiences and a greater eagerness to experiment produce a risk factor of higher involvement in substance use (Middendorff et al., 2012; Turiano et al., 2012; Benotsch et al., 2013). It also contradicts several findings regarding the use of multiple substances (e.g., illegal drug use, cigarette smoking, the non-medical use of prescription drugs), which found associations consistent with this assumption (e.g., Booth-Kewley and Vickers, 1994; Trull and Sher, 1994; Kornør and Nordvik, 2007; Terracciano et al., 2008; Turiano et al., 2012; Benotsch et al., 2013).

Respondents with higher levels of conscientiousness were less likely to report prior CE-drug use as well as a willingness to use CE drugs in the future. This is consistent with the supposition that conscientiousness serves as a protective factor against substance use (Middendorff et al., 2012; Turiano et al., 2012; Benotsch et al., 2013) and corroborates prior findings on a broad CE-drug measure (Middendorff et al., 2012) as well as many other substances such as alcohol consumption, cigarette smoking, prescription drug use, and illegal drug use (e.g., Trull and Sher, 1994; Kornør and Nordvik, 2007; Malouff et al., 2007; Terracciano et al., 2008; Turiano et al., 2012; Benotsch et al., 2013; Lackner et al., 2013; Atherton et al., 2014). This protective effect could be due to better self-regulation and persistence, which help to control impulses and delay the immediate gratifications of potentially health-damaging behaviors, while aiming for positive, long-term outcomes (such as long-term health) (Middendorff et al., 2012; Benotsch et al., 2013; Turiano et al., 2012). But also a greater ability to engage in systematic and organized behavior and consequently achieving better performance outcomes reduces the need to use CE drugs to compensate for the lack of such an ability (Middendorff et al., 2012). Furthermore, the increased tendency of conscientious individuals to follow norms might also decrease their likelihood to engage in behavior that many consider as morally objectionable (Sattler et al., 2013b; Dubljević et al., 2014; Schelle et al., 2014; Wiegel et al., 2015; Sattler, in press).

Our results also show no significant covariation between extraversion and our two CE-measures. This finding corroborates researchers' assumption that no such association should exist (Middendorff et al., 2012; Turiano et al., 2012) as well as the results of one study that found no association between CE-drug use and extraversion (Middendorff et al., 2012); at the same time some studies found that higher levels of extraversion can be associated with increased consumption of several substances (e.g., Kornør and Nordvik, 2007; Terracciano et al., 2008; Turiano et al., 2012; Atherton et al., 2014).

Agreeableness was neither significantly associated with our retrospective nor with the prospective CE-measure and thereby supports prior research on agreeableness and CE as well as the assumption that no such effect exists (Middendorff et al., 2012). For the use of other substances a negative association has been predicted (Booth-Kewley and Vickers, 1994; Turiano et al., 2012) and found (e.g., Kornør and Nordvik, 2007; Malouff et al., 2007; Terracciano et al., 2008; Turiano et al., 2012; Benotsch et al., 2013; Lackner et al., 2013), showing that agreeableness seems to have a protective effect.

It has also been shown that increased neuroticism leads to higher probabilities of prior CE-drug use and willingness to use CE drugs. Our results thus support the assumption that neuroticism is a risk factor for several kinds of substance use (Middendorff et al., 2012; Turiano et al., 2012; Benotsch et al., 2013). They also corroborate prior findings on a broad CE-measure (Middendorff et al., 2012) and other substances including prescription drugs, alcohol, nicotine, and illegal drugs (Sher et al., 2000; Malouff et al., 2007; Terracciano et al., 2008; Turiano et al., 2012; Benotsch et al., 2013; Lackner et al., 2013). One reason for these findings could be that neurotic individuals are less emotionally stable and more vulnerable to stress and therefore use these substances to cope with emotional distress (Kornør and Nordvik, 2007; Benotsch et al., 2013).

#### Associations between demographic controls and prior CE-drug use and willingness to engage in CE-drug use

With regard to the demographic controls, we found almost no significant differences regarding prior and potential future CE-drug use. While prior studies have shown mixed gender effects—generally finding no effect or a higher prevalence for males, but also that the purpose of use or the types of drugs used differ between sexes (e.g., McCabe et al., 2005; Rabiner et al., 2009, 2010; Weyandt et al., 2009; Ford and Ong, 2014; Singh et al., 2014; Ponnet et al., 2015; Wiegel et al., 2015)—our study found that women showed a higher willingness to use CE drugs, which is consistent with another German large-scale study (Hoebel et al., 2011). It has been assumed that such effects could indicate structural discrimination against women, namely that women need to work harder than men to rise in the hierarchy and at the same time often have twice or three times the amount of chores (work, children, and household) (Wiegel et al., 2015). This might increase the incentive for women to use such drugs as leverage in the job and to deal with their larger workload. Prior findings about the age-effect were indecisive (e.g., Maher, 2008; Terracciano et al., 2008; Benotsch et al., 2013; Ragan et al., 2013; Sattler and Wiegel, 2013; Sattler et al., 2013a; Ford and Ong, 2014; Singh et al., 2014; Wiegel et al., 2015), however, we found no age effect for both outcome variables. More years of education, however, were associated with a lower reporting of prior CE-drug use, which contradicts, for example, the study of Hoebel et al. (2011), which found no significant differences. One explanation could be that increased education is associated with greater knowledge about the limited efficiency of CE drugs. Additionally, for those with lower education, CE could be one means of compensating for lowered chances in the labor market or of dealing with potentially burdensome demands from their jobs. These assumptions have to be verified by future research. Respondents with potentially more monthly financial resources reported no elevated prior CE-drug use or willingness to do so in the future. In keeping with prior findings (Sattler and Wiegel, 2013; Sattler et al., 2014; Wiegel et al., 2015) and reasoning consistent with the Theory of Planned Behavior (Beck and Ajzen, 1991; Ouellette and Wood, 1998), we found a strong positive effect of prior CE-drug use on the willingness to consume such drugs in the future, indicating that many users have not only experimented once with these drugs but intend to continue using them (cf. Müller and Schumann, 2011). Users may have already made up their minds about their preferences or may be influenced by other factors such as a lack of self-control, sticking with their decisions out of habit and without further deliberation; they may have had positive experiences; or, in order to reduce potential cognitive dissonance, they may justify prior and continued drug consumption by ignoring negative information about CE-drug use or perceiving supporting information on a selective basis (Beck and Ajzen, 1991; Ouellette and Wood, 1998; Caviola et al., 2014; Wiegel et al., 2015).

### Limitations and strengths of the study and directions for future research

The awareness of potential limitations is important when interpreting our results. We will describe these together with the strengths of our study as well as suggest directions for future research:

1. Our response rate of 29.77% can be compared to similar studies (Bender et al., 2009; Schmich, 2015). But a considerable amount of invitees did not participate in our survey, which can reduce the external validity of the results if this non-response is selective. However, a comparison between the target population (information derived from German registry data) and our sample shows a high correspondence between socio-demographic characteristics, indicating limited problems of selectivity (see Methods section) (Diewald et al., 2014). Since we used a large representative population-based sample, our results might be more generalizable than the numerous small scale and non-representative samples in the field of CE-research and of research on the association between BFI and the non-medical use prescription drugs.
2. Our sample covers only the employed German population subject to social insurance contributions, hence it does not provide a full picture of the general population in Germany. However, our target population can be considered a large and important group in society. More research has been requested for our under-investigated target population (Greely et al., 2008; Ragan et al., 2013; Fitz et al., 2014; Schelle et al., 2014; Sattler, in press). This request is due to the repeated critique that most prior studies on factors influencing CE-drug use solely focused on students or other specific populations (Cutler, 2014; Ford and Ong, 2014; Wolff et al., 2014; Maier et al., 2015; Wiegel et al., 2015) and thus faced a limited generalizability.
3. We only investigated individuals from one country. CE-drug use as well as the association between CE and BFI might differ across countries, for example, due to varying regulations, social acceptance, advertisement, and the availability of CE drugs as well as (legal and illegal) alternative drugs that serve as substitutes (cf. Terracciano et al., 2008; Bell et al., 2013; N'Goran et al., 2014; Wiegel et al., 2015; Sattler, in press). Studies in other countries using a methodology similar to ours could provide insights about the cross-cultural generalizability of our results.
4. Several studies investigating the relationships between personality traits and (non-medical) drug (mis-)use investigate only a subset of Big Five traits (Terracciano et al., 2008). We assessed all five domains and thus can offer a more complete picture of these relationships. Since we could only employ a short scale of the Big Five Personality Traits Inventory (Gerlitz and Schupp, 2005; Dehne and Schupp, 2007; Hahn et al., 2012), only overall effects of the five higher level personality factors were explored (cf. Turiano et al., 2012). The BFI-S showed only moderate values concerning reliability, which has been documented in previous research (Dehne and Schupp, 2007). However, one would not expect high alpha values if each (single) item is supposed to capture a specific facet within a trait (Rammstedt, 2010). More importantly, however, the factor loadings were high and unambiguous, indicating that the BFI-S captures the underlying latent personality dimensions rather well. Still, future studies should investigate the full BFI or the NEO-PI-R (Berth and Goldschmidt, 2004; Ostendorf and Angleitner, 2004; Soto and John, 2009) to see if the results can be replicated with broader measures of personality and which lower level personality-facets of each trait are specifically relevant and predictive due to their higher specificity. It has been argued, however, that interpreting effects of domains is more basic and “combines information from several scales in meaningful ways and allows us to make more powerful inferences about personality traits and correlates that are not directly measured” (Costa and McCrae, 1995, p. 46). In addition to these instruments that target the Big Five traits, other personality trait inventories as well should be employed in future studies, such as the Minnesota Multiphasic Personality Inventory (MMPI) (e.g., Butcher, 2010), the 16PF Questionnaire (e.g., Cattell and Mead, 2008), or the Myers-Briggs Type Indicator (MBTI) (e.g., Myers et al., 1985).
5. Self-reporting CE-drug use can be seen as sensitive and thus may provoke drop-out, non-response, and underreporting^4^—especially if the anonymity of respondents is not guaranteed (Benotsch et al., 2013; Sattler, in press). This undoubtedly causes downward-biased prevalence estimates. In our telephone study, it was not possible to employ other measures such as testing hair, urine, or blood and contrast these results with self-report measures. But individuals might be reluctant to allow such tests (cf. N'Goran et al., 2014), which can thus also lead to distorted prevalence estimates due to selection bias. We did, however, inform the participants verbally and in writing about the measures to ensure the anonymity of their participation. Our results also show that item-nonresponse was considerably low (0.15% for prior CE-drug, and 1.89% for the willingness measure), which can be one indication of relatively low perceived sensitivity of the question resulting in a high confidentiality of answering (Sattler et al., 2013a). We calculated all models with raw data and after applying the multiple imputation procedure to test whether dropout and item-nonresponse affected our results. Our analysis show that the results are highly similar (see Tables S2, S3), which testifies to the robustness of our results.
6. Due to the low prevalence of CE-drug use, we only employed a lifetime prevalence measure of CE-drug use. However, future studies should distinguish periods of use (e.g., the 1-month or 12-months prevalence) more precisely, investigate frequency measures (e.g., to differentiate between regular use and one-time use), assess the dosage (e.g., to assess the severity of misuse), run drug-specific analyses, and investigate single- and poly-substance use (Turiano et al., 2012; Sattler and Wiegel, 2013; N'Goran et al., 2014; Sattler, in press) to further increase our understanding of the association between BFI and CE. As a second outcome variable, we probed the respondents about their potential future use of CE-drugs in general. Future studies could detail this by assessing willingness for specific situations and in specific contexts (e.g., in high stress situations). Such measure can be developed also in order to differentiate between behavioral willingness and behavioral intentions, e.g., in order to test the Prototype-Willingness Model (Gibbons et al., 1998a,b, 2009; Gerrard et al., 2006).
7. Another caveat of our research is associated with the use of cross-sectional data, implying that conclusions about the causal effect of personality are not warranted. Some scholars argue that drug use may cause changes in personality traits (Caspi et al., 2005; Normann and Berger, 2008; Kipke et al., 2010). Following this argument, the associations between the personality traits investigated and CE-drug use could be at least partially explained by drug-induced personality changes. To our knowledge no such research exists (yet) for CE-drug use, but it does for other substances (Bates and Pandina, 1991; Littlefield et al., 2010; Hicks et al., 2012; Hulka et al., 2015) and it could be assumed that such effects are possible for CE-drug use as well. On the other hand, it has been argued that personality traits are relatively stable entities (Costa and McCrae, 1988; Soldz and Vaillant, 1999; Caspi et al., 2005; Terracciano et al., 2006; Turiano et al., 2012) and it has been assumed that they might not change rapidly through CE-drug use (Metzinger, 2012; Wulf et al., 2012), whereas some research has shown trait changes for other substances after as little as a few weeks or months (Tang et al., 2009). One study has shown that personality still had predictive power if there was a long time-lag between the assessment of personality and substance use (Turiano et al., 2012). But this study also found that personality changes affected substance use. These findings call for more longitudinal research to assess the covariation of personality and CE-drug use or substance use in general over time (Sher et al., 2000; Turiano et al., 2012; N'Goran et al., 2014). However, our results show that the associations found between the willingness measure and the personality traits was similar for non-users and users. Thus, those who did not experience any potential personality changes from CE-drug use did not show a different willingness to use such drugs, which corroborates the effects on prior CE-drug use we found for personality traits. But still, our assessment of prior use did not cover the frequency, dosage, or duration of use, which can be seen as affecting substance-induced personality change. In addition, unmeasured confounder variables (such as genetic dispositions or social capital) could influence both personality and substance use, or personality could be influenced by these kinds of third variables, mediating their effect on substance use (Eysenck, 1999; Malouff et al., 2007; Schunck, 2014). Taken together and according to the reasoning of Malouff et al. (2007) on alcohol consumption, it is possible that (a) personality leads to CE-drug use, (b) CE-drug use leads to certain personality traits, (c) a third variable influences both, (d) personality mediates the effect of a third variable, or e) a combination of these effects is operating. Studies investigating these possibilities should also investigate variables that might influence the relationship between personality traits and CE-drug use, such as stress, social pressure, etc. (Francis, 1996; Benotsch et al., 2013). Data allowing such investigations do not currently exist.

This exploratory study investigated how prior and future CE-drug use and the Big Five traits are associated. To better understand its findings and those of earlier studies on the Big Five traits and various substances used for CE and other purposes, future research should put more emphasis on developing a coherent theoretical model. To test this model, more highly elaborated and fine-grained measures should be employed in order to challenge the robustness of our findings and obtain a more thorough comprehension of the relationship.

### Conclusion

This large-scale study is based on a random sample of employees in Germany and shows that the use of prescription drugs to augment cognitive performance among healthy individuals is an empirical reality. However, this behavior is less widespread than had been anticipated by many scholars and media reports. But the significantly greater willingness to use CE drugs compared to the lifetime prevalence may be indicative of a possible increase in CE-drug use in the future. Still, the extent to which willingness to use CE drugs translates into actual behavior must be addressed in longitudinal studies. It remains to be discussed which threshold of willingness and prevalence justifies further prevention and regulation means. At the very least, a non-negligible number of individuals already risks side-effects, long-term health consequences, and the involvement of the criminal justice system by using often non-efficient pharmaceutical agents. These individuals may also contribute to pressuring others to use such drugs, to increasing healthcare costs, and to other issues discussed in the ethics debate (e.g., CE-drug use in relation to the authenticity of users, fairness, or social inequality). This study increases our understanding of potential psychological factors that hamper or foster the use of CE drugs. We found that high levels of conscientiousness were associated with decreased retrospective/prospective consumption, while high levels of neuroticism increased it. Such insights about personality profiles could be used to inform the development of treatment approaches tailored to these profiles in order to minimize health problems (Booth-Kewley and Vickers, 1994; Terracciano et al., 2008; Conrod et al., 2011). Another approach would be to develop interventions to promote beneficial personality traits (e.g., increasing conscientiousness) and thereby support a positive change toward health-related behaviors in general (Magidson et al., 2014; Hudson and Fraley, 2015). However, less risky options for enhancing one's cognitive performance (such as sufficient sleep, seeking support, meditation, physical exercise) should be promoted for those who want or must enhance their performance (e.g., Bostrom and Sandberg, 2009; Dresler et al., 2013; Maier and Schaub, 2015; Wiegel et al., 2015).

## Author contributions

Wrote the Paper: SS, RS. Analyzed the data: RS.

### Conflict of interest statement

The authors declare that the research was conducted in the absence of any commercial or financial relationships that could be construed as a potential conflict of interest. The funders did not influence any interpretations or force the research team to produce biased results. The views expressed do not necessarily reflect the policies of the funder. The authors did not receive any research support from public or private actors in the pharmaceutical sector. The authors do not have any competing financial interests.

## Footnotes

^1^In our study, we focus on prescription drugs that seem to dominate the current debate (e.g., Farah et al., 2004; Glannon, 2008; Greely et al., 2008; Racine and Forlini, 2010; Smith and Farah, 2011; Ragan et al., 2013; Schelle et al., 2014; Sattler, in press). Among these are drugs such as prescription stimulants (e.g., methylphenidate), antidementives (e.g., donepezil), and antidepressants (e.g., fluoxetine). The term “cognitive enhancement” generally encompasses the use of various potential enhancement means such as illegal drugs (e.g., cocaine), over-the-counter drugs (e.g., guarana), drugs naturally synthecized in the body (e.g., insulin), but also non-substance-based means such as mental training, sleep, brain stimulation (e.g., Bostrom and Sandberg, 2009; Dresler et al., 2013).

^2^In line with a developmental framework (e.g., McLarnon et al., 2012), individuals in different stages of life might have different demands or propensities to use CE drugs (e.g., due to different peer exposure, risk preferences, or stressful periods). We therefore additionally tested quadratic and cubic functions of the age effect. Because no such effects were found, we do not show the results.

^3^Again, quadratic and cubic functions of the age effect were not significant.

^4^Over-reporting might occur in rare cases, but it is assumed to be less likely in our context since most people in prior studies voiced moral objections to using CE-drugs Sattler et al., 2013a,b; Schelle et al., 2014; Wiegel et al., 2015.
