# Supplementary material for: Associations Between the Big Five Personality Traits and the Non-Medical Use of Prescription Drugs for Cognitive Enhancement
Source: Front Psychol. 2016 Jan 5;6:1971. doi: 10.3389/fpsyg.2015.01971 (PMC4700267; doi:10.3389/fpsyg.2015.01971)
Supplement: Supplementary file 1 [file Table1.docx]

**SUPPLEMENTS**

**Table S1.** Factor loadings of the BFI-S items based on principal component factor analysis with a varimax rotation (Eigenvalues >1) with non-imputed data (number of observations: N=6,407).

|  | **Factor 1 E** | **Factor 2 C** | **Factor 3 N** | **Factor 4 O** | **Factor 5 A** | **Uniqueness** |
| --- | --- | --- | --- | --- | --- | --- |
| 1. Imagination [O] | -.206 | -.021 | -.027 | -**.725** | -.058 | -.428 |
| 2. Ideas [O] | -.273 | -.242 | -.108 | -**.589** | -.085 | -.501 |
| 3. Artistic [O] | -.052 | -.039 | -.050 | -**.712** | -.134 | -.468 |
| 4. Thorough [C] | -.018 | -**.772** | -.051 | -.051 | -.124 | -.383 |
| 5. Efficient [C] | -.123 | -**.758** | -.094 | -.09 | -.023 | -.393 |
| 6. Lazy [C –] | -.104 | **-.551** | -.038 | -.172 | -.101 | -.645 |
| 7. Talkative [E] | -**.783** | -.14 | -.019 | -.161 | -.135 | -.322 |
| 8. Sociable [E] | -**.773** | -.112 | -.004 | -.161 | -.107 | -.353 |
| 9. Reserved [E –] | **-.719** | -.090 | -.179 | -.101 | -.223 | -.383 |
| 10. Kind [A] | -.132 | -.268 | -.070 | -.095 | -**.696** | -.413 |
| 11. Forgiving [A] | -.068 | -.075 | -.104 | -.215 | -**.516** | -.666 |
| 12. Rude [A –] | -.047 | -.031 | -.075 | -.117 | **-.759** | -.402 |
| 13. Relaxed [N –] | -.037 | -.227 | **-.663** | -.104 | -.140 | -.477 |
| 14. Nervous [N] | -.090 | -.076 | -**.754** | -.046 | -.002 | -.415 |
| 15. Worried [N] | -.001 | -.203 | -**.717** | -.045 | -.043 | -.441 |

Source: LEEP-B3, own computations.

“–“ in column 1 indicates that the items have a reverse polarity. Bold figures indicate the highest loading of an item. The wording of the items was as follows: “I see myself as someone who…” for **Openness to experience (O)**: 1. “…has an active imagination.”, 2. “…is original, comes up with new ideas.”, 3. “…values artistic experiences.”; for **Conscientiousness (C)**: 4. “…does a thorough job.”, 5. “…does things effectively and efficiently.”, 6. “…tends to be lazy.” [–]; for **Extraversion (E)**: 7. “…is communicative, talkative.”, 8. “…is outgoing, sociable.”, 9. “…is reserved.” [–]; for **Agreeableness (A)**: 10. “…is considerate and kind to others.”, 11. “…has a forgiving nature.”, 12. “…is sometimes somewhat rude to others.” [–]; for **Neuroticism (N)**: 13. “…is relaxed, handles stress well.” [–], 14. “…gets nervous easily.” 15. “…worries a lot.” Items were rated on a scale from “agree completely” (1) to “disagree completely” (6).
