# Supplementary material for: Associations Between the Big Five Personality Traits and the Non-Medical Use of Prescription Drugs for Cognitive Enhancement
Source: Front Psychol. 2016 Jan 5;6:1971. doi: 10.3389/fpsyg.2015.01971 (PMC4700267; doi:10.3389/fpsyg.2015.01971)
Supplement: Supplementary file 2 [file Table2.docx]

**SUPPLEMENTS**

**Table S2.** Logistic regression models to assess associations of the BFI-S and socio-demographic controls with prior CE-drug use (Model 1) and the willingness to use CE drugs (Model 2 and 3) with non-imputed data.

|  | **Model 1**  **Prior CE-drug use** | |  | **Model 2**  **Willingness to use CE drugs** | |  | **Model 3**  **Willingness to use CE drugs** | |
| --- | --- | --- | --- | --- | --- | --- | --- | --- |
|  | *OR* | 95% *CI* |  | *OR* | 95% *CI* |  | *OR* | 95% *CI* |
| Openness to experience | 01.113^***^ | [0.958,1.294] |  | 01.027^***^ | [0.942,1.121] |  | 01.012^***^ | [0.924,1.108] |
| Conscientiousness | 00.774^***^ | [0.672,0.891] |  | 00.819^***^ | [0.756,0.888] |  | 00.842^***^ | [0.774,0.915] |
| Extraversion | 01.068^***^ | [0.922,1.238] |  | 01.061^***^ | [0.976,1.154] |  | 01.055^***^ | [0.968,1.150] |
| Agreeableness | 00.947^***^ | [0.826,1.086] |  | 00.942^***^ | [0.869,1.022] |  | 00.945^***^ | [0.867,1.030] |
| Neuroticism | 01.390^***^ | [1.181,1.636] |  | 01.305^***^ | [1.198,1.422] |  | 01.259^***^ | [1.152,1.375] |
| Male | 00.877^***^ | [0.631,1.218] |  | 00.799^***^ | [0.669,0.955] |  | 00.802^***^ | [0.666,0.966] |
| Age in years | 00.997^***^ | [0.980,1.015] |  | 01.007^***^ | [0.997,1.017] |  | 01.008^***^ | [0.998,1.018] |
| Education in years | 00.949^***^ | [0.896,1.005] |  | 00.988^***^ | [0.959,1.019] |  | 00.997^***^ | [0.966,1.029] |
| Gross monthly earnings in Euro | 01.000^***^ | [1.000,1.000] |  | 01.000^***^ | [1.000,1.000] |  | 01.000^***^ | [1.000,1.000] |
| Prior CE-drug use |  |  |  |  |  |  | 16.347^***^ | [11.839,22.572] |
| Constant | 00.076^***^ | [0.023,0.246] |  | 0.114^***^ | [0.062,0.211] |  | 00.080^***^ | [0.042,0.150] |
| Log pseudolikelihood | -798.537 | |  | -1,992.617 | |  | -1,846.752 | |
| Pseudo *R*² | 0.027 | |  | 0.020 | |  | 0.092 | |
| Number of observations | 6,058 | |  | 5,967 | |  | 5,967 | |

Source: LEEP-B3, own computations.

OR = Odds Ratios. CI = 95% confidence intervals in parentheses (based on robust standard errors). Log pseudolikelihood and Pseudo R² are averaged across imputed datasets.

* *p* < .05, ** *p* < .01, *** *p* < .001.
