# Supplementary material for: Associations Between the Big Five Personality Traits and the Non-Medical Use of Prescription Drugs for Cognitive Enhancement
Source: Front Psychol. 2016 Jan 5;6:1971. doi: 10.3389/fpsyg.2015.01971 (PMC4700267; doi:10.3389/fpsyg.2015.01971)
Supplement: Supplementary file 3 [file Table3.docx]

**SUPPLEMENTS**

**Table S3.** Logistic regression models to assess associations of the BFI-S and socio-demographic controls with and the willingness to use CE drugs including interactions with Prior CE-drug use (Model 1: with non-imputed data and Model 2: with imputed data with 20 imputation).

|  | **Model 1**  **Willingness to use CE drugs**  (Non-imputed data) | |  | **Model 2**  **Willingness to use CE drugs**  (Imputed data) | |
| --- | --- | --- | --- | --- | --- |
|  | *OR* | 95% *CI* |  | *OR* | 95% *CI* |
| Openness to experience | 001.012^***^ | [0.920,1.114] |  | 001.001^***^ | [0.911,1.100] |
| Conscientiousness | 000.832^***^ | [0.763,0.908] |  | 000.823^***^ | [0.755,0.896] |
| Extraversion | 001.068^***^ | [0.976,1.169] |  | 001.075^***^ | [0.983,1.177] |
| Agreeableness | 000.933^***^ | [0.854,1.020] |  | 000.922^***^ | [0.845,1.007] |
| Neuroticism | 001.276^***^ | [1.164,1.400] |  | 001.280^***^ | [1.168,1.403] |
| Male | 000.820^***^ | [0.676,0.995] |  | 000.818^***^ | [0.675,0.990] |
| Age in years | 001.008^***^ | [0.997,1.019] |  | 001.006^***^ | [0.995,1.017] |
| Education in years | 001.007^***^ | [0.974,1.041] |  | 001.008^***^ | [0.976,1.041] |
| Gross monthly earnings in Euro | 001.000^***^ | [1.000,1.000] |  | 001.000^***^ | [1.000,1.000] |
| Prior CE-drug use | 100.034^***^ | [9.913,1009.485] |  | 131.630^***^ | [12.998,1332.972] |
| Openness to experience *x* Prior CE-drug use | 000.934^***^ | [0.682,1.280] |  | 000.919^***^ | [0.672,1.256] |
| Conscientiousness *x* Prior CE-drug use | 001.185^***^ | [0.884,1.587] |  | 001.184^***^ | [0.886,1.582] |
| Extraversion *x* Prior CE-drug use | 000.896^***^ | [0.662,1.213] |  | 000.898^***^ | [0.663,1.216] |
| Agreeableness *x* Prior CE-drug use | 001.281^***^ | [0.903,1.818] |  | 001.297^***^ | [0.917,1.833] |
| Neuroticism *x* Prior CE-drug use | 000.855^***^ | [0.630,1.159] |  | 000.857^***^ | [0.633,1.160] |
| Male *x* Prior CE-drug use | 000.875^***^ | [0.417,1.836] |  | 000.859^***^ | [0.415,1.777] |
| Age in years *x* Prior CE-drug use | 001.010^***^ | [0.972,1.049] |  | 001.010^***^ | [0.973,1.049] |
| Education in years *x* Prior CE-drug use | 000.885^***^ | [0.776,1.009] |  | 000.867^***^ | [0.761,0.988] |
| Gross monthly earnings in Euro *x* Prior CE-drug use | 001.000^***^ | [1.000,1.000] |  | 001.000^***^ | [1.000,1.000] |
| Constant | 000.068^***^ | [0.035,0.133] |  | 000.071^***^ | [0.037,0.136] |
| Log pseudolikelihood | -1,840.726 | |  | -1,948.434 | |
| Pseudo *R*² | 0.095 | |  | 0.097 | |
| Number of observations | 5,967 | |  | 6,454 | |

Source: LEEP-B3, own computations. OR = Odds Ratios. CI = 95% confidence intervals in parentheses (based on robust standard errors). Log pseudolikelihood and Pseudo R² are averaged across imputed datasets.

* *p* < .05, ** *p* < .01, *** *p* < .001.
